# CRISPR-Cas-Mediated Phage Resistance Enhances Horizontal Gene Transfer by Transduction

**DOI:** 10.1128/mBio.02406-17

**Published:** 2018-02-13

**Authors:** Bridget N. J. Watson, Raymond H. J. Staals, Peter C. Fineran

**Affiliations:** a Department of Microbiology and Immunology, University of Otago, Dunedin, New Zealand; b Laboratory of Microbiology, Wageningen University, Wageningen, The Netherlands; c Bio-Protection Research Centre, University of Otago, Dunedin, New Zealand; University of California, San Francisco; Harvard Medical School

**Keywords:** CRISPR-Cas, bacteriophages, genomic islands, horizontal gene transfer, plasmids, transduction

## Abstract

A powerful contributor to prokaryotic evolution is horizontal gene transfer (HGT) through transformation, conjugation, and transduction, which can be advantageous, neutral, or detrimental to fitness. Bacteria and archaea control HGT and phage infection through CRISPR-Cas (clustered regularly interspaced short palindromic repeats–CRISPR-associated proteins) adaptive immunity. Although the benefits of resisting phage infection are evident, this can come at a cost of inhibiting the acquisition of other beneficial genes through HGT. Despite the ability of CRISPR-Cas to limit HGT through conjugation and transformation, its role in transduction is largely overlooked. Transduction is the phage-mediated transfer of bacterial DNA between cells and arguably has the greatest impact on HGT. We demonstrate that in *Pectobacterium atrosepticum*, CRISPR-Cas can inhibit the transduction of plasmids and chromosomal loci. In addition, we detected phage-mediated transfer of a large plant pathogenicity genomic island and show that CRISPR-Cas can inhibit its transduction. Despite these inhibitory effects of CRISPR-Cas on transduction, its more common role in phage resistance promotes rather than diminishes HGT via transduction by protecting bacteria from phage infection. This protective effect can also increase transduction of phage-sensitive members of mixed populations. CRISPR-Cas systems themselves display evidence of HGT, but little is known about their lateral dissemination between bacteria and whether transduction can contribute. We show that, through transduction, bacteria can acquire an entire chromosomal CRISPR-Cas system, including *cas* genes and phage-targeting spacers. We propose that the positive effect of CRISPR-Cas phage immunity on enhancing transduction surpasses the rarer cases where gene flow by transduction is restricted.

## IMPORTANCE

The generation of genetic diversity through acquisition of DNA is a powerful contributor to microbial evolution and occurs through transformation, conjugation, and transduction. Of these, transduction, the phage-mediated transfer of bacterial DNA, is arguably the major route for genetic exchange. CRISPR-Cas adaptive immune systems control gene transfer by conjugation and transformation, but transduction has been mostly overlooked. Our results indicate that CRISPR-Cas can impede, but typically enhances the transduction of plasmids, chromosomal genes, and pathogenicity islands. By limiting wild-type phage replication, CRISPR-Cas immunity increases transduction in both phage-resistant and -sensitive members of mixed populations. Furthermore, we demonstrate mobilization of a chromosomal CRISPR-Cas system containing phage-targeting spacers by generalized transduction, which might partly account for the uneven distribution of these systems in nature. Overall, the ability of CRISPR-Cas to promote transduction reveals an unexpected impact of adaptive immunity on horizontal gene transfer, with broader implications for microbial evolution.

## INTRODUCTION

CRISPR-Cas (clustered regularly interspaced short palindromic repeats–CRISPR-associated proteins) systems confer adaptive immunity in prokaryotes. These systems are composed of CRISPR arrays, consisting of short repeats separated by spacer sequences derived from invading nucleic acids, and the CRISPR-associated (*cas*) genes. CRISPR-Cas systems function in three main stages: acquisition (or adaptation), where new invader-derived (e.g., plasmid or phage) spacers are incorporated into the CRISPR array; expression, when the CRISPR array is expressed and processed into short crRNAs (CRISPR RNAs); and, finally, interference, whereby Cas-crRNA ribonucleoprotein complexes bind and degrade complementary foreign nucleic acids (for reviews, see references 1, 2, 3, 4, and 5). Since the demonstration of an antiviral role for CRISPR-Cas in 2007 (6), much has been revealed of the exquisite mechanism of these systems (3), leading to their exploitation in various applications (7).

In addition to the role of CRISPR-Cas systems in phage resistance, they inhibit conjugation and transformation, thereby limiting HGT (8, 9). Because HGT significantly influences bacterial evolution, most visibly through the dissemination of antibiotic resistance and virulence determinants (10–14), the ability of CRISPR-Cas to impede the acquisition of mobile genetic elements has been considered an evolutionary downside (15). However, the acquisition or maintenance of other genetic elements can have associated costs (16, 17), and, in such cases, CRISPR-Cas immunity would be beneficial (18). Therefore, the influence of CRISPR-Cas on HGT has remained a matter of debate. Recently, it was shown that there is no detectable influence of CRISPR-Cas on HGT over evolutionary timescales (19), suggesting that the inhibitory effects of bacterial adaptive immunity on HGT are somehow balanced.

Of the three major forms of HGT, transduction is likely to be the most influential globally. However, the impact of CRISPR-Cas on transduction has been mostly overlooked, with the exception of a single study (20). Transduction involves the phage-mediated transfer of nonviral genes and is classified as either specialized or generalized (21, 22). Temperate phages cause specialized transduction when the prophage excises imprecisely from the host chromosome and accidentally packages bacterial genes flanking the integration site, which can be transferred, upon infection of a new host. Since specialized transduction mobilizes only sequences adjacent to the prophage site, its contribution to HGT is limited. In contrast, generalized transduction occurs when either virulent or temperate phages make errors upon DNA packaging by mistakenly incorporating only bacterial DNA (either chromosomal or plasmid) (11, 13, 21). This results in viral populations of predominantly infectious phages and rare subpopulations of transducing particles. Upon host cell binding, transducing particles inject bacterial DNA, which is degraded or recombined with the bacterial genome. Since the generalized transducing particle contains no phage DNA, viral progeny are not produced, but the more abundant neighboring infectious phages can proceed through normal propagation (21).

The host range of transducing phages can be broad, infecting different bacterial classes (10, 23), and in natural environments, transduction occurs between diverse bacteria and ecosystems (24–26). Transduction occurs in about one in 10^7^ to 10^9^ infections (13), although single-cell studies suggest a higher frequency (27). Given the abundance of phages and the high rate of bacterial infection (≥10^30^ phages on Earth and ~10^25^ infections/s) (14), generalized transduction is frequent and widespread and has a profound impact on genetic exchange in prokaryotes (>10^16^ gene transfer events/s) (13, 28). In this study, we addressed the effects of CRISPR-Cas on HGT of plasmids, chromosomal loci, and a pathogenicity island resulting from generalized transduction and show that the source of the spacer, targeting either phage or transduced DNA, determines whether transduction is enhanced or inhibited. Since spacers are more commonly acquired from phages, we propose that the dominant effect of CRISPR-Cas on transduction is enhancement. We show that strains with spacers targeting phages provide viral protection at the population level, enhancing transduction of both phage-sensitive and phage-resistant bacteria. Finally, generalized transduction also enabled the dissemination of the CRISPR-Cas systems themselves.

## RESULTS

### CRISPR-Cas interferes with transduction of plasmids and chromosomal genes.

**FIG 1:**
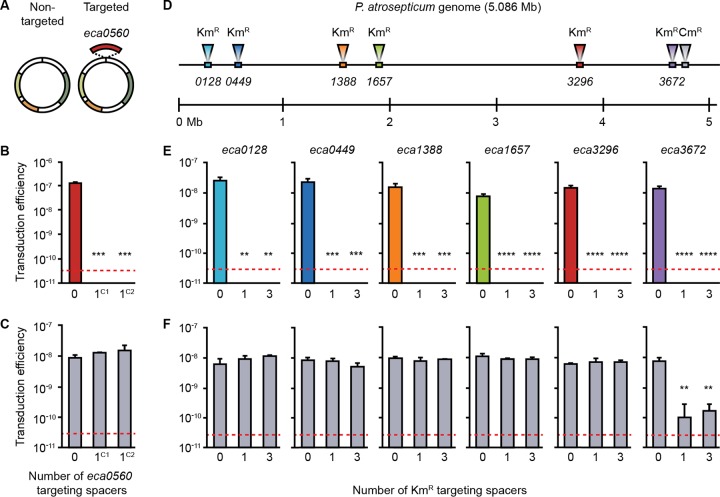
CRISPR-Cas can inhibit transduction of plasmids and chromosomal loci. (A) Transducing phage φTE was grown on strains carrying either a control vector (Non-targeted) or a vector with a copy of the *eca0560* gene (Targeted). (B and C) The targeted (B) and nontargeted (C) vectors were transduced into strains with either no spacers targeting *eca0560* (0; strain ΔHAI2) or one spacer targeting the *eca0560* gene in either CRISPR1 (1^C1^; strain PIM06) or CRISPR2 (1^C2^; strain PIM17). For additional data from controls, see Fig. S1. (D) Map of the *P. atrosepticum* genome, indicating the locations of 6 Km^r^-marked chromosomal loci and of the secondary Cm^r^ marker in the *cas* operon in the six strains (strains PCF83 to PCF88). (E and F) The transducing phage, φTE, was grown on these six strains and used to transduce each Km^r^ marker (E) and Cm^r^ marker (F) into strains either lacking spacers targeting the Km^r^ gene (0; strain ΔHAI2) or with one (1; strain PIM18) or three (3; strain PIM28) spacers in CRISPR1 targeting the Km^r^ gene. Data are shown as the mean number of transductants/PFU + standard deviation (SD) (*n* = 3). The dashed line in each panel represents the limit of detection (one transductant across the replicates). Statistical significance was calculated using one-way analysis of variance (ANOVA) and Dunnett’s multiple-comparison test, comparing strains with targeting spacers to the control with no targeting spacers (**, *P* ≤ 0.01; ***, *P* ≤ 0.001; ****, *P* ≤ 0.0001). Additional data from the controls are shown in Fig. S1 and S2.

*Pectobacterium atrosepticum* contains a type I-F CRISPR-Cas system with three CRISPR arrays (CRISPR1 to CRISPR3) that naturally acquire spacers under physiological conditions (29). We previously isolated strains that had acquired spacers that inhibited plasmid uptake by conjugation and transformation (30). To investigate whether the type I system of *P. atrosepticum* inhibited generalized transduction of plasmids, we used generalized transducing phage φTE, which is a member of the *Myoviridae* (31, 32). Phage φTE was grown on *P. atrosepticum* cells containing either a control plasmid or a plasmid carrying a targeted gene (*eca0560*) (Fig. 1A). Since generalized transduction results from errors occurring during DNA packaging, the vast majority of the resulting viral particles are wild-type (WT) phages, while a small subpopulation consists of transducing particles, containing various regions of chromosomal DNA or the plasmids. The resulting phage progeny were used to infect a control strain and derivatives with a single additional newly acquired *eca0560*-targeting spacer in either CRISPR1 or CRISPR2 (strains are listed in Table S1 in the supplemental material) (30). Transduction of either plasmid occurred at ~10^−7^ to 10^−8^ transductants/plaque forming unit (PFU) (Fig. 1B and C). However, a single *eca0560*-targeting spacer in either array interfered with plasmid transduction by >10^3^-fold (limit of detection), whereas the nontargeted control was unaffected (Fig. 1B and C; further data from controls are shown in Fig. S1 in the supplemental material). These experiments show the potential of CRISPR-Cas to inhibit generalized transduction and support an earlier study using the type III CRISPR-Cas system of *Staphylococcus epidermidis* to prevent transduction of plasmids that were engineered to contain phage sequence (20).

10.1128/mBio.02406-17.1 FIG S1  Confirmation of plasmid transduction using PCR and phage infectivity assays. (A) φTE transduced a vector with a copy of the *eca0560* gene (Targeted) and a control vector (Non-targeted). (B) A representative group of transductants plus the recipient (R) strains, carrying no targeting spacers (0; ΔHAI2 control) or one spacer targeting *eca0560* in either CRISPR1 (strain PIM06) or CRISPR2 (strain PIM17), and the donor (D) strain were screened for the presence of the targeted plasmid (with a predicted product size of 2,321 bp) or nontargeted plasmid (with a predicted product size of 241 bp) using colony PCR. No PCR screening was performed when no transductants were detected. Product sizes were compared with those of the Invitrogen 1 kb-plus DNA marker. (C) The φTE lysates produced on either strain, carrying the targeted or nontargeted vector, were titrated on the three recipient strains, including a strain without any *eca0560*-targeting spacers (0; ΔHAI2 control) and strains with one spacer targeting the *eca0560* gene in CRISPR1 (1^C1^, strain PIM06) or CRISPR2 (1^C2^, strain PIM17). Data are shown as the mean PFU ml^−1^ + SD (*n* = 3). Download FIG S1, TIF file, 8.9 MB. Copyright © 2018 Watson et al. 2018 Watson et al. This content is distributed under the terms of the Creative Commons Attribution 4.0 International license.

10.1128/mBio.02406-17.6 TABLE S1  Bacterial strains and plasmids used in this study. Download TABLE S1, DOCX file, 0.02 MB. Copyright © 2018 Watson et al. 2018 Watson et al. This content is distributed under the terms of the Creative Commons Attribution 4.0 International license.

Next, we examined whether CRISPR-Cas inhibits generalized transduction of chromosomal loci. We used six strains marked with kanamycin resistance (Km^r^) at different chromosomal locations and a secondary (chloramphenicol resistance [Cm^r^]) marker disrupting the *cas* operon (Fig. 1D). The donor *cas* mutation excluded potential CRISPR-Cas effects prior to transduction and provided an additional phenotype to differentiate transductants from donors. φTE transduced all six loci with similar levels of efficiency (~10^−8^ transductants/PFU) into the wild-type *P. atrosepticum* (see control data in Fig. 1E; see also Fig. S2). When recipients contained either one or three spacers targeting the Km^r^ gene in each of the six chromosomal locations, no transductants were detected (Fig. 1E). This effect was transduction specific, because φTE infected all strains with equal levels of efficiency (Fig. S2A and B). The inhibition was sequence specific, because transfer of the untargeted Cm^r^ marker was unaffected (Fig. 1F). One exception was observed when the Cm^r^ marker was located close (~17 kb) to one targeted locus, showing CRISPR-Cas-dependent interference with linked cotransduced genes (Fig. 1F; *eca3672*)—potentially due to the processive nuclease-helicase activity of the Cas2-3 fusion protein (33). Therefore, CRISPR-Cas interfered in a sequence-specific manner with the transduction of plasmids and chromosomal loci and cotransduced genetically linked DNA.

10.1128/mBio.02406-17.2 FIG S2  Confirmation of the transduction of chromosomal loci using PCR and phage infectivity assays. (A) Map of the *P. atrosepticum* genome with the locations of Km^r^-marked chromosomal loci and the secondary Cm^r^ marker shown. (B) The φTE lysates made on the six strains with different marked chromosomal loci were titrated on the three recipient strains with zero (0; ΔHAI2 control), one (1; strain PIM18), or three (3; strain PIM28) Km^r^-targeting spacers. (C) A representative group of transductants plus the recipient strain (denoted with an "R") lacking any Km^r^-targeting spacers (0; ΔHAI2 control) and the donor strains (denoted with a "D") were screened for the presence of the Km^r^ marker in the correct loci. (D) A representative group of transductants plus the recipient strains with zero (0; ΔHAI2 control), one (1; strain PIM18), or three (3; strain PIM28) Km^r^-targeting spacers and the donor strain were screened for the presence of the Cm^r^ marker. No PCR screening was performed when no transductants were detected. Product sizes were compared with that of the Invitrogen 1 kb-plus DNA marker. Download FIG S2, TIF file, 18.5 MB. Copyright © 2018 Watson et al. 2018 Watson et al. This content is distributed under the terms of the Creative Commons Attribution 4.0 International license.

### CRISPR-Cas can inhibit pathogenicity island transduction.

**FIG 2:**
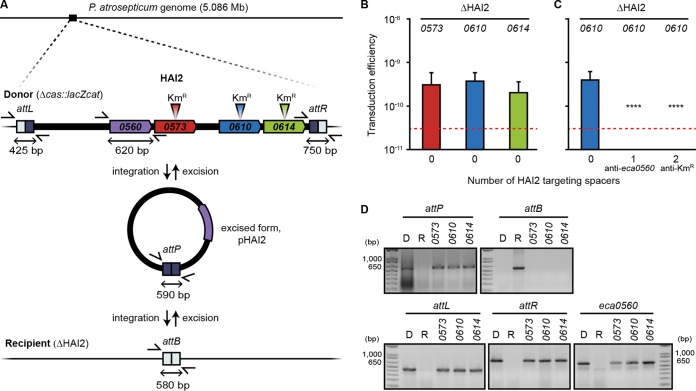
CRISPR-Cas can inhibit pathogenicity island transduction. (A) Maps of the HAI2 pathogenicity island with the relevant loci present in the donor strains and of the recipient strain lacking the island are shown. (B) HAI2, marked by a Km^r^ gene in one of three different loci (*eca0573*, *eca0610*, and *eca0614*) was transduced into an islandless strain (0; strain ΔHAI2) (*n* = 11). (C) The *eca0610* marked strain was used to transduce the island into a control strain lacking spacers targeting the island (0; strain ΔHAI2) or into recipients with one spacer targeting the HAI2 gene, *eca0560* (1; strain PIM06), or two spacers targeting Km^r^ (2; strain PIM31) (*n* = 9). (D) A representative transductant from each of the marked loci in the island plus the islandless recipient strain (R) and a representative donor strain, PCF90 (D), were screened for the entire pathogenicity island and the formation of new strains. Loci detected included the excised island form *attP*, the absence of the island (*attB*), regions flanking the island (*attL* and *attR*), and an island gene (*eca0560*). Product sizes were compared with those of an Invitrogen 1 kb-Plus DNA marker. Data are shown as the mean number of transductants/PFU + SD. The dashed line represents the limit of detection (one transductant across the replicates). Statistical significance was calculated using one-way ANOVA and Dunnett’s multiple-comparison test, comparing strains with targeting spacers to the control with no targeting spacers (C) (****, *P* ≤ 0.0001). Additional data from controls are shown in Fig. S3.

Genomic islands often carry genes important for fitness, including those encoding antibiotic resistance and virulence determinants (17). *P. atrosepticum* contains an ~98-kb island (HAI2) encoding a polyketide phytotoxin important for virulence *in planta* (34). HAI2, a member of the integrative and conjugative elements (ICEs), excises from the chromosome and circularizes (35) (Fig. 2A), but no mechanism of transfer has been demonstrated. Because φTE is ~142 kb, it theoretically could package the complete ~98-kb HAI2 island. Indeed, φTE mediated the transfer of HAI2 from donors marked in three different island loci (*eca0573*, *eca0610*, and *eca0614*; Km^r^) to both the wild-type recipients and the islandless recipients (Fig. 2A and B; see also Fig. S3). Analysis of the integration sites and other unique loci revealed that the entire HAI2 island was transferred and recombined precisely into the recipients (Fig. 2D; see also Fig. S3). We predicted that CRISPR-Cas could inhibit transduction of genomic islands. In agreement, HAI2 was not transferred into strains with either a spacer targeting an island gene (*eca0560*) or two spacers targeting a Km^r^ marker elsewhere on the island (Fig. 2C). φTE infected all recipients equally (Fig. S3), showing that the inhibition was a CRISPR-specific transduction effect. We recently showed that populations of *P. atrosepticum* can acquire spacers targeting HAI2 (29), which can trigger complete island loss or mosaicism (36). Here, we now demonstrate that such spacers targeting the island can then impede the reintroduction of the HAI2 pathogenicity island when it is disseminated by φTE-mediated transduction.

10.1128/mBio.02406-17.3 FIG S3  Transduction of the HAI2 pathogenicity island was confirmed using PCR and infectivity assays. (A) Map of HAI2 with 3 Km^r^ markers in different loci. (B) A representative group of transductants were screened for the presence of the marker in the correct loci. The donor (denoted with a "D") and recipient lacking HAI2 (denoted with an "R") were included as controls. (C) φTE was grown on three strains, each with a Km^r^ marker in a different locus in HAI2, and titrated on the recipient strain lacking the island (0; ΔHAI2). (D) The lysate grown on the *eca0610* Km^r^ marked strain was also titrated on the recipient strain lacking the island (0; ΔHAI2) and strains containing one spacer targeting the island gene, *eca0560* (1; strain PIM06), or two spacers targeting the Km^r^ gene (2; strain PIM31). (E and F) The 3 Km^r^ markers in HAI2 were then transduced into a wild-type recipient containing HAI2 (E) and titrated on the wild-type recipient strain (F). Transduction efficiency data are shown as numbers of transductants/PFU. All data are shown as means + SD (*n* = 3). The red dashed line indicates the limit of detection (one transductant across the replicates). Download FIG S3, TIF file, 8.5 MB. Copyright © 2018 Watson et al. 2018 Watson et al. This content is distributed under the terms of the Creative Commons Attribution 4.0 International license.

### Phage resistance conferred by CRISPR-Cas enhances transduction.

We have shown that spacers targeting transduced DNA enable CRISPR-Cas to limit the transduction of plasmids, chromosomal genes, and genomic islands. Despite using sensitive nested-PCR techniques, we could not detect spacer acquisition from transduced DNA (Fig. S4), which was unsurprising since only ~10^−8^ cells underwent transduction and since adaptation in *P. atrosepticum* is rare under laboratory conditions (29). Although the level of adaptation was below our detection limit, the global estimate of 10^25^ phage infections/s indicates that acquisition undoubtedly occurs during transduction. Previously, spacers were detected in archaeal genomes that matched nonmobile chromosomal regions of other archaea (37) and it was proposed that these might be derived from transduction (37). However, it is possible that these spacers were acquired through other routes (29, 38). Irrespective of their source, we have shown that spacers matching transduced DNA inhibit transduction.

10.1128/mBio.02406-17.4 FIG S4  The level of spacer acquisition during transduction was below the limit of detection. (A) Schematic of nested PCR showing primers used and product size for rounds 1 and 2 to amplify CRISPR1 from the strain carrying a HAI2-targeting spacer (PIM20). (B) PIM20 was diluted and combined with cells lacking the island-targeting spacer (ΔHAI2), creating samples with increasing proportions of PIM20 cells (samples 1 to 11; 12 = blank [no DNA control]) in a total of 10^10^ cells. Nested PCR was carried out on the pooled cells as shown in panel A. (C) Schematic showing nested PCR primers and product sizes to amplify CRISPR1 from ΔHAI2 cells to screen for the integration of the HAI2-targeting spacer. (D) An example of round 2 PCR on HAI2 cells following island transduction (lanes 1 to 3, results of one replicate, the experiment was performed in triplicate). Product sizes were compared with those of the Invitrogen 1 kb-plus DNA marker. Download FIG S4, TIF file, 8.2 MB. Copyright © 2018 Watson et al. 2018 Watson et al. This content is distributed under the terms of the Creative Commons Attribution 4.0 International license.

**FIG 3:**
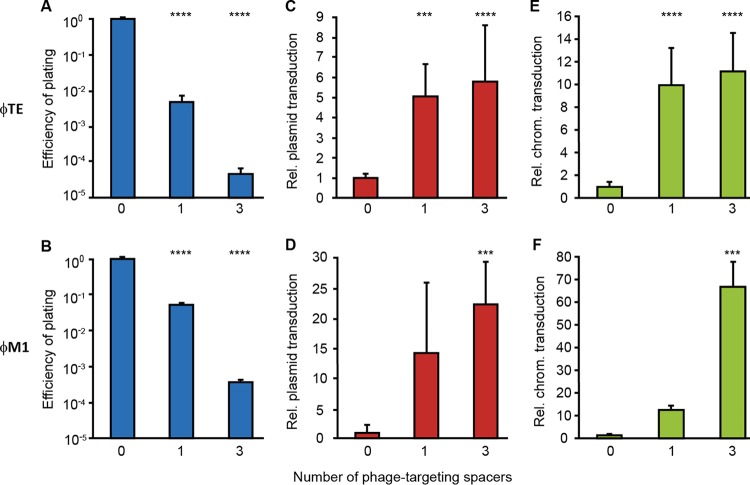
Phage targeting by CRISPR-Cas enhances generalized transduction. (A) φTE was titrated on the control strain (0; strain SCRI1043 [WT]) and on strains with one φTE-targeting spacer (1; strain PCF190) or three φTE-targeting spacers (3; strain PCF188), and the efficiency of plating was defined as the φTE-targeting strain titer/WT titer. (B) Similarly, φM1 was titrated on the control strain (0; strain SCRI1043 [WT]) and on strains with one φM1-targeting spacer (1; strain PCF254) or three φM1-targeting spacers (3; strain PCF256) and the efficiency of plating was defined as the φM1-targeting strain titer/WT titer. (C to F) A plasmid (pTRB30) (C and D) and a chromosomal (chrom.) marker (donor strain PCF88) (E and F) were transduced into the control strain and the anti-φ strains. Relative (Rel.) levels of transduction were determined as phage-targeting strain transduction efficiency/WT transduction efficiency. Data are shown as means + SD (*n* = 3) (A, B, and F); (*n* = 6) (D) and (*n* = 9) (C and E). Statistical significance was calculated using one-way ANOVA and Dunnett’s multiple-comparison test, comparing strains with targeting spacers to the control with no targeting spacers (***, *P* ≤ 0.001; ****, *P* ≤ 0.0001).

Because spacers matching phage sequences are more commonly observed than spacers matching chromosomal sequences (39), and since transduction efficiencies are low (Fig. 1 and 2), we estimated that spacer acquisition from phages would occur at rates substantially (~10^6^-fold to 10^9^-fold) higher than from transduced DNA. In addition, in nature, acquisition of phage-targeting spacers imposes a strong selective advantage compared with acquisition of spacers from transduced DNA. Because transducing particles typically represent a rare subpopulation of wild-type phages, new transductant bacteria are at risk of subsequent phage infection—reducing the establishment and maintenance of horizontally transferred genes. Since phage-derived spacers protect bacteria from infection (6), we hypothesized that the canonical role of CRISPR-Cas in phage resistance would enhance HGT by increasing the survival of transductants. To test this hypothesis, we isolated *P. atrosepticum* strains that had acquired spacers targeting two different phages, φTE and φM1 (an ~44-kb generalized transducing *Podoviridae* unrelated to φTE [32, 40]). Experiments performed with strains with one or three phage-targeting spacers resulted in an up to >10^4^-fold reduction in phage infectivity (Fig. 3A and B). In support of our hypothesis, when plasmids or chromosomal loci were transduced into the φTE-resistant strains, the abundance of transductants was ~5-fold or ~10-fold higher, respectively, than that of the phage-sensitive control strain (Fig. 3C and E). Moreover, an even greater elevation in transduction was observed for φM1, with >15-fold and >60-fold increases in plasmid and chromosomal transfer, respectively (Fig. 3D and F). Therefore, by eliciting phage resistance, CRISPR-Cas can enhance the maintenance of genes transferred by transduction by two distinct phages.

### CRISPR-Cas enhances population-level transduction by reducing phage abundance.

**FIG 4:**
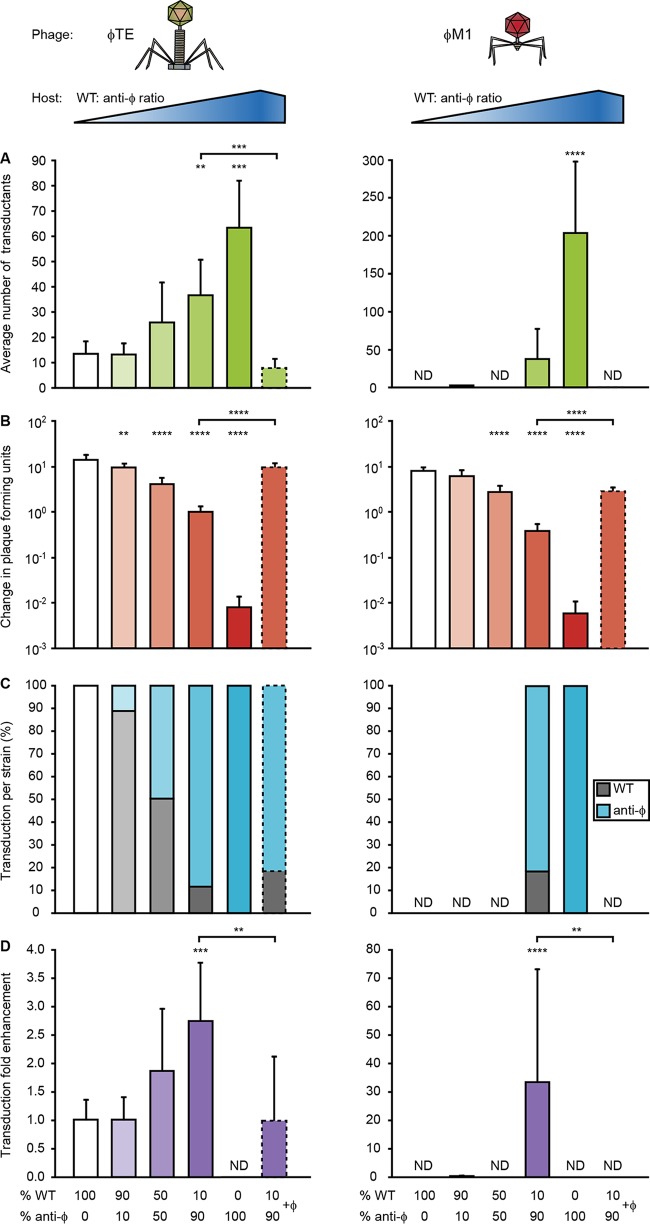
CRISPR-Cas enhances transduction at the population level by reducing phage abundance. A phage-sensitive WT strain (PCF326) and phage-resistant anti-φ strains (PCF332 [3× anti-φTE] and PCF400 [3× anti-φM1]) were combined in different proportions (WT/anti-φ ratios, 100:0, 90:10, 50:50, 10:90, and 0:100) and infected with a transducing lysate at an MOI of 1. (A and B) Average numbers of transductants (A) and changes in PFUs (B) were determined for two phages, φTE and φM1. ND, not detected. (C) The percentages of WT transductants (gray bars) and anti-φ transductants (blue bars) were calculated for each assay. (D) The number of transductants in the WT background was adjusted to its proportion in each assay to calculate the transduction fold enhancement. In panels A to D, the experiments using a WT/anti-φ ratio of 10:90 were complemented with phages to mimic a phage burst (bars with dashed lines). Data are shown as means + SD (*n* = 6). Statistical significance was calculated using one-way ANOVA and Dunnett’s multiple-comparison test, comparing strains with targeting spacers (90:10, 50:50, 10:90, and 0:100) to the control with no targeting spacers (100:0). The 10:90 phage complementation data were compared to the 10:90 data using an unpaired *t* test (**, *P* ≤ 0.01; ***, *P* ≤ 0.001; ****, *P* ≤ 0.0001).

One explanation for enhanced transduction of CRISPR phage-resistant strains (abbreviated as "anti-φ") could be their ability to reduce the effects of lytic wild-type phages by decreasing their abundance. Therefore, we measured φTE- and φM1-mediated transduction of phage-sensitive (WT) and anti-φ populations and quantified viral abundance (Fig. 4A and B). The increased transduction in the anti-φ strain was accompanied by a >1,000-fold reduction in phage titer relative to the phage-sensitive WT strain (Fig. 4A and B; compare 100% WT [white] with 100% anti-φ [dark green/red]). We reasoned that the reduced phage epidemic protected the anti-φ transductants from further viral infection. Therefore, we predicted that, by reducing subsequent phage bursts, CRISPR-Cas could increase transduction of phage-sensitive bacteria within populations containing anti-φ members. To test this, anti-φ bacteria were cocultured with the phage-sensitive WT in different proportions (WT/anti-φ ratios, 100:0, 90:10, 50:50, 10:90, and 0:100) and cells were exposed to transducing phages for a round of phage infection. In agreement with our prediction, the overall number of transductants rose as the anti-φ proportion increased (Fig. 4A).

It was possible that the elevated transduction was due to increased numbers of resulting anti-φ transductants, due to their higher initial levels. However, in these cocultures, the levels of enhancement of transduction were similar for the two strains (i.e., the number of transductants for each strain reflected the initial ratio for each strain) (Fig. 4C and S5). Accounting for the proportions of each strain, transduction into the phage-sensitive strain benefited from the presence of the anti-φ strain as its abundance in the population increased (Fig. 4D).

10.1128/mBio.02406-17.5 FIG S5  The initial proportions of WT and anti-φ strains in the mixed-culture assays. The initial proportions of WT and anti-φ strains in the mixed-culture assays were determined by colony counting; data are shown as percentages. The initial proportions were compared to the proportion of transductants (Fig. 4C) using an unpaired *t* test (*P* ≥ 0.5 for each assay). Data are shown as means (*n* = 6). Download FIG S5, TIF file, 3.1 MB. Copyright © 2018 Watson et al. 2018 Watson et al. This content is distributed under the terms of the Creative Commons Attribution 4.0 International license.

There was an inverse correlation between transduction efficiency and the final virus abundance (i.e., fewer phages remained in populations with more of the anti-φ strain, and those conditions exhibited greater numbers of transductants) (Fig. 4B). To confirm that the high final phage abundance was responsible for the lower transduction levels, we took cocultures with a high anti-φ proportion (WT/anti-φ ratio, 10:90) and, by spiking in WT phages, simulated the phage epidemic present in cultures with lower transduction efficiencies. Consistent with the model, enhanced transduction was abolished, resulting in levels similar to those seen with the phage-sensitive monoculture that failed to suppress the phages (Fig. 4A and B; 100% WT). These results demonstrate that anti-φ strains enhance transduction by reducing the subsequent wild-type viral load, protecting the entire population, which can include phage-sensitive neighbors.

### Escape phages enable further gene transfer via transduction.

**FIG 5:**
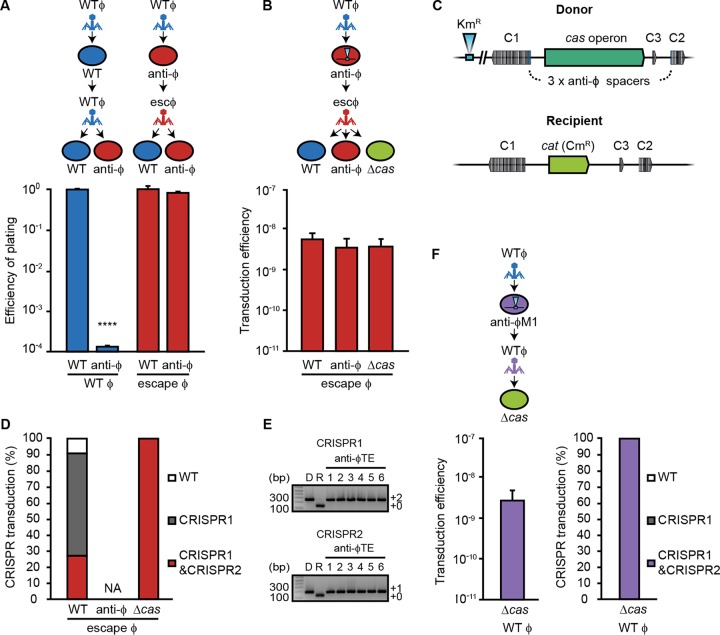
Escape phage populations enable further gene transfer via transduction and the transfer of CRISPR-Cas systems. (A) Efficiency of plating assays were performed on the WT or anti-φ strain (3× anti-φTE), using WT φTE (blue) or escape φTE phages (red) generated following growth on a strain with 3× anti-φTE spacers (PCF288). (B) The escape φTE phages were used to transduce a CRISPR-Cas-linked chromosomal marker (*eca3672*::Km) into WT and anti-φ strains and a strain lacking the *cas* operon (Δ*cas*). (C) Schematic showing the strains used in the experiments whose results are presented in panels B and F. The donor had the *cas* operon, a linked Km^r^ marker, and three phage-targeting spacers. The recipient strain lacked the *cas* operon and phage-targeting spacers. C1, CRISPR1; C2, CRISPR2; C3, CRISPR3. (D) Efficiency of CRISPR-Cas transduction. CRISPR arrays of transductants were screened for the transfer of phage-targeting spacers (white bars, original WT CRISPR; gray bars, two phage-targeting spacers in CRISPR1; red bars, three spacers in CRISPR1 and CRISPR2). The means are shown (*n* = 3). NA, not assessed. (E) Representative PCR gel showing the transferred phage-targeting spacers in six Δ*cas* transductants, in CRISPR1 and CRISPR2. Product sizes were compared with that of the Invitrogen 1 kb-plus DNA marker. (F) The *cas* operon and phage-targeting spacers were transduced from 3× anti-φM1 (PCF287) into the Δ*cas* mutant. Data are shown as means + SD (WT and anti-φ strains, *n* = 3; Δ*cas* mutant, *n* = 6). Statistical significance was calculated using an unpaired *t* test (****, *P* ≤ 0.0001).

The ability of anti-φ strains to reduce phage abundance (Fig. 4B) suggests that subsequent rounds of transduction might initially be decreased. However, we predicted that the emergence of escape phages insensitive to the anti-φ spacers would enable further transduction. Indeed, following infection, φTE and φM1 escape phages were produced by anti-φ strains at a frequency of ~10^−3^ to 10^−4^ (Fig. 3A and B). We examined the φTE escape phages further. These progeny φTE phage populations had escaped CRISPR interference and, as such, efficiently infected the anti-φ strain compared with WT φTE (Fig. 5A). Next, we investigated the ability of these escape phages to continue further rounds of transduction. The escape φTE phages were grown on bacteria originating from the first round of transduction that were resistant to WT φTE due to the presence of anti-φTE spacers (Fig. 5A). The φTE escape phages transduced a Km^r^ chromosomal marker from the anti-φTE strains into the WT strain and an anti-φTE strain with similar efficiencies (Fig. 5B). These results demonstrate that cycles of phage escape and transduction can continue even after anti-φ strains emerge, provided that the initial viral population is large enough to contain escape mutants. These observations are consistent with the phage-host CRISPR coevolutionary dynamics documented in both the laboratory and the natural environment where escaping phages emerged and then the bacteria acquired further spacers in response (41, 42).

### Phages can mobilize CRISPR-Cas resistance via transduction.

The ability of phages to transduce chromosomal regions and the role of CRISPR-Cas in enhancing this process—even to phage-sensitive neighbors—led us to query whether the CRISPR-Cas systems themselves were transferrable by transduction. Indeed, we showed that φTE was able to mobilize chromosomal regions of ~100 kb—big enough to encompass a CRISPR-Cas system (Fig. 2). Furthermore, in the experiments shown in Fig. 1, we demonstrated that φTE mobilized a genomic region encompassing CRISPR arrays (detected by Km^r^ in *eca3672* and a Cm^r^ marker in the *cas* operon). To examine if CRISPR-Cas systems could be mobilized by transduction, we used φTE escape populations that were grown on strains containing a Km^r^ marker close to the CRISPR-Cas locus (Fig. 5B and C). The φTE escape transducing population mobilized the Km^r^ marker into both WT and anti-φTE strains (Fig. 5B). When the chromosomal marker was transduced into the phage-sensitive strain, most of the transductants had also acquired the phage-targeting spacers in CRISPR1 and/or CRISPR2, due to the linkage of the arrays with the Km^r^ marker (Fig. 5C and D). This showed that the φTE escape population can efficiently transduce the entire CRISPR-Cas locus containing the 3× anti-φTE spacers into strains that were previously phage sensitive. Therefore, escape phages can disseminate CRISPR arrays that contain anti-φ spacers by generalized transduction.

It was also of interest to determine if an entire CRISPR-Cas system could be transferred by transduction into strains that lack *cas* genes and therefore have no functional adaptive immunity. We demonstrated that the entire CRISPR-Cas system, including the *cas* operon and three associated CRISPR arrays with the antiphage spacers, could be transduced into strains lacking the *cas* operon and phage resistance (Fig. 5B to E). Since φTE escape phages can transduce CRISPR arrays carrying spacers targeting the WT φTE genome, the resulting transductants become immunized against WT phages (Fig. 5A). In a similar fashion, φTE can mobilize CRISPR-Cas loci containing spacers targeting another phage (φM1) (Fig. 5F). This shows that transducing phages can move entire CRISPR-Cas systems and spacers in arrays. Therefore, phages may alter, via generalized transduction, the specific phage resistance profile of their hosts and the dissemination of CRISPR-Cas systems.

## DISCUSSION

By expanding on an earlier study (20), we have demonstrated that the transduction of plasmids, chromosomal loci, and genomic islands can be limited by CRISPR-Cas interference. Therefore, it is clear that in addition to conjugation and transformation (8, 9), the third major route of HGT, transduction, can also be inhibited by CRISPR-Cas. However, due to the infrequency of spacers acquired during transduction versus from phages, the dominant role of CRISPR-Cas in transduction is unlikely to be that of inhibition. We show that the acquisition of phage-targeting spacers enabled CRISPR-Cas to protect bacteria from phage infection, limited the effect of wild-type phages, and increased the generation of transductants. This positive influence of CRISPR-Cas on HGT was unexpected, and we hypothesize that it might be one route to counteract the proposed evolutionary downside of adaptive immunity on the acquisition of beneficial genes (15).

Short-term laboratory experiments have shown that one response to this downside appears to be selection for strains that have inactivated adaptive immunity when there is a strong selective pressure for HGT (15). For example, when *Staphylococcus epidermidis* containing a spacer matching an antibiotic resistance plasmid was forced to acquire the plasmid, the isolates had mutations that inactivated CRISPR-Cas immunity (15). Additionally, the presence of CRISPR-Cas inversely correlated with acquired antibiotic resistance in multiple isolates of *Enterococcus faecalis* (43). Despite the evolutionary downside observed in these examples, results of a large computational study led to the conclusion that any effect of CRISPR-Cas on impeding HGT was undetectable over longer evolutionary times and, hence, that support for the idea of an evolutionary downside was lacking (19). The positive effects of CRISPR-Cas-mediated phage resistance on HGT via transduction that we observed may provide one route to rebalance gene flow and enable HGT and adaptive evolution in the presence of CRISPR-Cas immunity. Although we showed that transduction can be inhibited by CRISPR-Cas, the fitness benefits associated with virus protection, and the rates of dissemination of genetic material by transduction, argue that the selective pressure for acquisition of phage-targeting spacers likely outweighs that of the infrequent acquisition of spacers targeting transduced DNA.

Our experiments most closely represent a migration of phages into a population or an increase in numbers of phages due to a burst in part of the population. It is more challenging to predict the outcome following multiple rounds of phage infections, due to the complexities of the dynamics of phage and host numbers, the evolution of phage resistance, escape phage frequencies, and the ability to transduce CRISPR-Cas immunity itself. It is possible that, over multiple rounds of infection, CRISPR adaptation events could eventually lead to the extinction of transducing phages (44) and eliminate the potential to generate transducing particles until escape phages emerge. However, in the environment, phages and their hosts typically coexist. In agreement, we show that, following a round of infection, escape phages are detectable and are capable of continuing transduction. Therefore, we predict that the population might cycle between periods of enhanced transduction with anti-φ strains, leading to phage suppression, followed by the appearance of escape phages (41, 45). Understanding these dynamics will be interesting and will require a combination of experimental and theoretical methods to quantify the different host and phage populations during cycles of infection and transduction. Irrespective of the exact dynamics, in the presence of phages, phage-resistant transductants are expected to outcompete any sensitive bacteria in the community.

CRISPR-Cas systems are present on chromosomes, plasmids, genomic islands, and even phages and are sporadically distributed between different bacterial taxa (46–48). In agreement, it has been suggested that some are disseminated horizontally by mechanisms such as conjugation (46). We show that transduction can mobilize chromosomally located CRISPR-Cas systems, in addition to plasmids and genomic islands. Therefore, CRISPR-Cas systems on plasmids and genomic islands are also likely to be transferred by transduction. The ability of some transducing phages to transfer genetic material between bacterial genera (10) might explain how highly related defense systems can be present in phylogenetically distinct bacteria. In addition to transduction of the *cas* operon, CRISPR arrays and phage-targeting spacers were transferred. The ability of phages to transfer spacers that provide resistance to other phages is predicted to generate bacterial hosts that are permissive to the transducing phage but with reduced competition from other phages. Transducing phages can also mobilize innate immune defenses. For example, in *P. atrosepticum*, φTE escape phages transduced the ToxIN toxin-antitoxin/abortive infection system, which provided resistance to wild-type φTE phages and other phages, such as φM1 (31).

Surprisingly, anti-φ strains not only enhanced the transduction of themselves but also of phage-sensitive bacteria by reducing the subsequent wild-type phage abundance in the population. An interesting possibility raised by our results is that, in populations with both phage-resistant and phage-sensitive strains, transduction of CRISPR-Cas phage resistance into phage-sensitive recipients might be enhanced. Therefore, it appears to represent a complex dynamic between CRISPR-Cas-mediated phage resistance and transduction. Since phages are estimated to promote >10^16^ gene transfer events/s (13, 28), their role in the spread of CRISPR-Cas systems is likely to be significant. Finally, from a biotechnological perspective, the ability to program cells as more-robust recipients during transduction could also provide new approaches for enhancing genetic modification of bacteria.

## MATERIALS AND METHODS

### Bacterial strains, plasmids, and growth conditions.

Bacterial strains and plasmids used in this study are given in Table S1 in the supplemental material. *P. atrosepticum* SCRI1043 (49) was grown at 25°C and *E. coli* at 37°C in lysogeny broth (LB) at 180 rpm or on LB agar (LBA) plates containing 1.5% (wt vol^−1^) agar. When required, media were supplemented with chloramphenicol (Cm; 25 µg ml^−1^) or kanamycin (Km; 50 µg ml^−1^). Bacterial growth was measured in a Jenway 6300 spectrophotometer at 600-nm optical density (OD_600_).

### Phage lysate preparation and titration.

The transducing phages, φTE (31) (genome size of ~142 kb) and φM1 (32, 40), were stored in phage buffer (10 mM Tris-HCl [pH 7.4], 10 mM MgSO_4_, 0.01% [wt vol^−1^] gelatin). Lysates were made by serially diluting phages in phage buffer, adding the mixture to 100 µl of a mixture consisting of *P. atrosepticum* culture (pregrown in 5-ml LB overnight) and 4 ml top LBA (0.35% [φTE] and 0.5% [φM1] agar), and pouring the result onto LBA plates. Plates were incubated at 25°C overnight, plaques were counted, and the titer was determined. Top agar from plates with almost confluent lysis was harvested with 3 ml of phage buffer, subjected to vortex mixing with 500 µl chloroform (saturated with NaHCO_3_) for approximately 2 min, and centrifuged at 2,219 × *g* for 20 min at 4°C. The supernatant was collected, 100 µl of chloroform was added, and lysates were stored at 4°C. Titers of phages were determined as described above, typically resulting in high-titer stocks (5 × 10^10^ to 4 × 10^11^ PFU ml^−1^).

### Transduction assays.

Duplicate 6-ml cultures of recipient strains were grown overnight and combined. The cultures were diluted to an OD_600_ of 2, and 10 ml (total, 1 × 10^10^ CFU) was pelleted and resuspended in 1 ml LB. Phage lysates were adjusted to 1 × 10^11^ PFU ml^−1^, and 100 µl (1 × 10^10^ PFU) was added at a multiplicity of infection (MOI) of 1. Transductions were incubated statically for 15 min at 25°C, and then 9 ml of LB (25°C) was added and the tubes were shaken for 45 min at 90 rpm on a slight angle at 25°C. Cells were pelleted at 2,219 × *g* for 9 min at room temperature, the supernatant was removed, and the pellet was resuspended in 10 ml LB. This step was repeated three times to remove excess phages, and the resulting material was finally resuspended in 1 ml LB. A 100-µl sample was plated onto LBA with the appropriate antibiotics, and the rest was pelleted and plated onto the same medium. Plates were incubated at 25°C for up to 5 days, and transductants were counted. Transduction efficiency was calculated as the number of transductants per PFU. Control samples of phage lysate and recipients were plated on the same antibiotics to check for contamination and spontaneous resistance, respectively, and no colonies were detected in any experiment. For infectivity controls, φTE titers were calculated for all lysates on their respective recipient strains to rule out differences in infection that might influence the determination of transduction efficiency (e.g., resulting from a receptor mutation).

### Plasmid transduction.

*P. atrosepticum* Δcas (Cm^r^; strain PCF79 [50]) was made electrocompetent as described previously (36) and transformed with purified pTRB30 and pPF189 plasmids. φTE lysates were prepared on PCF79 transformed with pTRB30 and on PCF79 transformed with pPF189. The lysates were used to transduce the ΔHAI2, PIM06, and PIM17 strains. The CRISPR array spacer content of the PIM strains used in this study was determined by colony PCR and sequencing performed with primers for CRISPR1 (PF174 and PF175), CRISPR2 (PF176 and PF177), or CRISPR3 (PF178 and PF179) as described previously (30). All oligonucleotides used in this study are listed in Table S2. Plasmid transduction was verified by antibiotic resistance (gain of Km^r^) and by PCR for the plasmid by using primers PF209 and PF210 (see Fig. S1A and B in the supplemental material). An infectivity control verified that all recipients had the same φTE sensitivity (Fig. S1C). Colonies were also checked for Cm sensitivity, and the results indicated that the colonies were not the original PCF79 strains used for lysate production. Mock lysates (containing no phages) were harvested from strain PCF79 containing either pTRB30 or pPF189 as described earlier but with phage buffer and no phage in the overlays. Mock lysates were used in transductions and did not facilitate plasmid transduction.

10.1128/mBio.02406-17.7 TABLE S2  Oligonucleotides used in this study. Download TABLE S2, DOCX file, 0.01 MB. Copyright © 2018 Watson et al. 2018 Watson et al. This content is distributed under the terms of the Creative Commons Attribution 4.0 International license.

### Transduction of chromosomal loci.

φTE lysates were prepared on six strains (PCF83 to PCF88) and used in transduction assays with strains ΔHAI2, PIM18, PIM28, and PIM86 as recipients with selection performed on Km (to detect Km^r^ transfer) or Cm (to detect Cm^r^ control transfer). Phage lysate and recipient controls were also plated as described for the transduction assay. Representative transductants (where obtained) were patched onto LBA with Km and LBA with Cm. Putative transductants were verified by PCR for each marked locus with PF1212 (binds to marked insertion) in combination with the following primers: PF1575 (*eca0128*), PF1573 (*eca0449*), PF1457 (*eca1388*), PF1577 (*eca1657*), PF1459 (*eca3296*), and PF1460 (*eca3672*) (Fig. S2C). To verify the transduction of the *cat* gene, primers PF432 and PF433 were used (Fig. S2D). As a control transduction, φTE lysates were prepared using the wild-type *P. atrosepticum* strain (i.e., resulting in no marked transducing particles). When this was used in control transductions, no transductants (Km^r^) were obtained.

### Transduction of HAI2.

To check HAI2 transfer, lysates of φTE were prepared on three strains (PCF89 to PCF91) and used in transduction assays performed with the ΔHAI2 strain or the wild-type strain as the recipient. Since homologous recombination positively affects transduction efficiency, transduction to islandless recipients occurred at a 10-fold-lower frequency (~0.5 × 10^−9^ transductants/PFU) (Fig. 2B) than was seen with wild-type recipients (~0.5 × 10^−8^ transductants/PFU). Putative HAI2 transductants were verified by plating on LBA with Km or Cm and by using PCR to amplify features of the various strains. PCR was performed with primer pairs as follows: *cas1* with PF390 and PF391, *cat* (Cm^r^ gene) with PF432 and PF433, *attP* (circularized pHAI2) with PF1225 and PF1226, *attB* (the HAI2 insertion site) with PF1227 and PF1228, *attL* (island border regions) with PF1227 and PF1226, *attR* with PF1225 and PF1228, and, finally, a gene in the island, *eca0560*, with PF1446 and PF1447. To check CRISPR-Cas inhibition of transfer, φTE prepared on PCF90 was used in transduction assays with strains ΔHAI2, PIM06, and PIM31 as recipients.

### Transduction with antiphage strains.

Transduction assays with antiphage strains were carried out using overnight cultures at an OD_600_ adjusted to 2 (1 × 10^9^ CFU), and the cultures were resuspended in 1 ml LB in 50-ml tubes. Phages were added at an MOI of 1 (1 × 10^9^ PFU). Assays with φTE were incubated for 15 min statically, followed by 45 min of shaking, before a 100-µl sample and then the remaining amount were plated. Reaction mixtures used for assays with φM1 were incubated for a total of 20 min with shaking, prior to plating onto media with antibiotics.

### Efficiency of plating and efficiency of transduction assays.

Strains that had acquired φTE- and φM1-targeting spacers were isolated as previously described (51). The value representing the efficiency of plating was defined as the titer of the phage-resistant test strains (PCF188, PCF190, PCF193, PCF254, and PCF256)/the titer of the control strain (SCRI1043). The transducing lysates were prepared on PCF88 and PCF79 with pTRB30 plasmid, and the chromosomal marker and plasmid were transduced into recipients. Efficiency of transduction was determined by calculation of the test transduction efficiency/control (SCRI1043) efficiency.

### Mixed-culture transduction assays.

Monocultures of the phage-sensitive WT (PCF326) strain and phage-resistant CRISPR (PCF332 [anti-φTE] and PCF400 [anti-φM1]) strains were grown overnight and combined in WT/anti-φ ratios of 100:0, 90:10, 50:50, 10:90, and 0:100, and 1 × 10^9^ cells were used for each assay. Assays were performed with six replicates for both φTE and φM1. Phage lysate was added at an MOI of 1, and cells were shaken for 1 h, in 1 ml LB, before being plated onto LBA with selection. Transductants were patched onto LBA containing Nal or Sm to identify the host strain. Colony forming units (CFUs) were determined by taking initial and final samples (10 µl), plating onto LBA, and patching 100 colonies onto LBA containing Nal or Sm. Total PFU counts were determined by adding an aliquot (10 µl) of culture to LB containing chloroform. PFU fold change was calculated as final PFU/initial PFU. Transduction fold enhancement for WT strains was calculated as follows: (number of transductants/average number of transductants in the 100:0 assay)/proportion of WT cells in the assay.

### Transduction of CRISPR by escape phages.

Transduction assays were performed as described above (see "Transduction with antiphage strains"). The transduction of CRISPR-Cas into the Δ*cas* strain was identified by plating transductants onto LBA carrying Km to select for transfer of the chromosomal marker. Transductants were then patched onto LBA with or without Cm to screen for loss of Cm^r^, indicating that the *cas* genes had been transferred. PCR screening of CRISPR arrays to detect the transduction of spacers was performed using primers PF174 and PF175 for CRISPR1 and primers PF176 and PF177 for CRISPR2 (Table S2).

### Nested PCR to screen for spacer acquisition.

Nested PCR was performed using primers PF1730 and PF1732 (round 1) followed by PF1730 and PF1733 (round 2). PCR products from round 1 were purified using an Illustra GFX PCR DNA and gel band purification kit (GE Healthcare) and were used as the round 2 template. To test the level of detection provided by PCR, the strains (PIM20 and ΔHAI2) were grown overnight. The PIM20 strain was serially diluted and combined with the ΔHAI2 strain at appropriate proportions based on OD_600_ values to create samples with 10^0^ to 10^10^ PIM20 cells in a pool of 10^10^ total cells. An aliquot of each sample was used for PCR. Island transduction was carried out as described above (see "Transduction of HAI2"), using a donor with a Km^r^ marker in *eca0573* (PCF89). Following transduction, pooled culture was used as the template for round 1 PCR.
