# Supplementary figures and images for: CRISPR-Cas-Mediated Phage Resistance Enhances Horizontal Gene Transfer by Transduction
Source: mBio. 2018 Feb 13;9(1):e02406-17. doi: 10.1128/mBio.02406-17 (PMC5821089; doi:10.1128/mBio.02406-17)

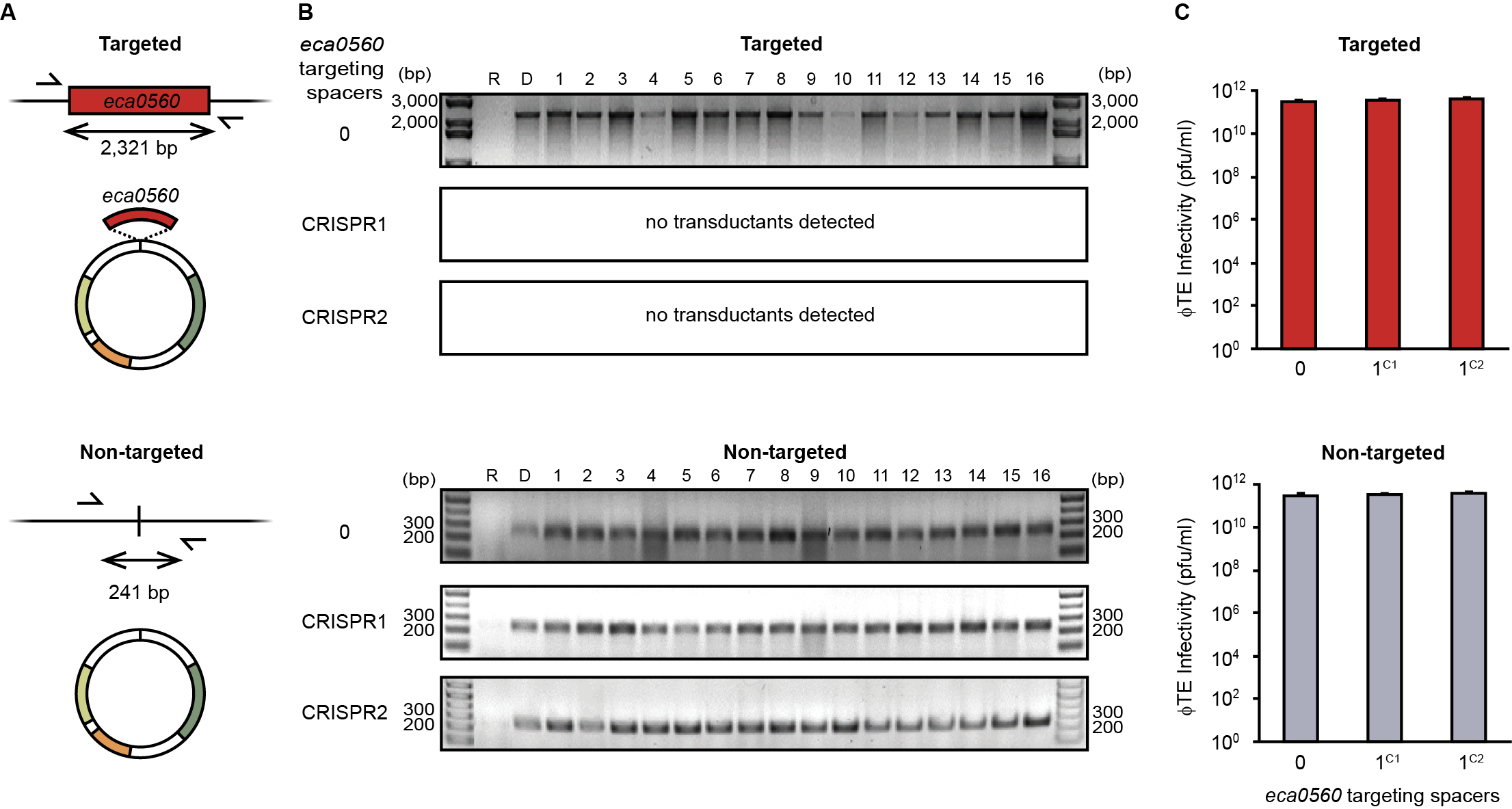

Supplement: FIG S1 [file mbo001183732sf1.tif]

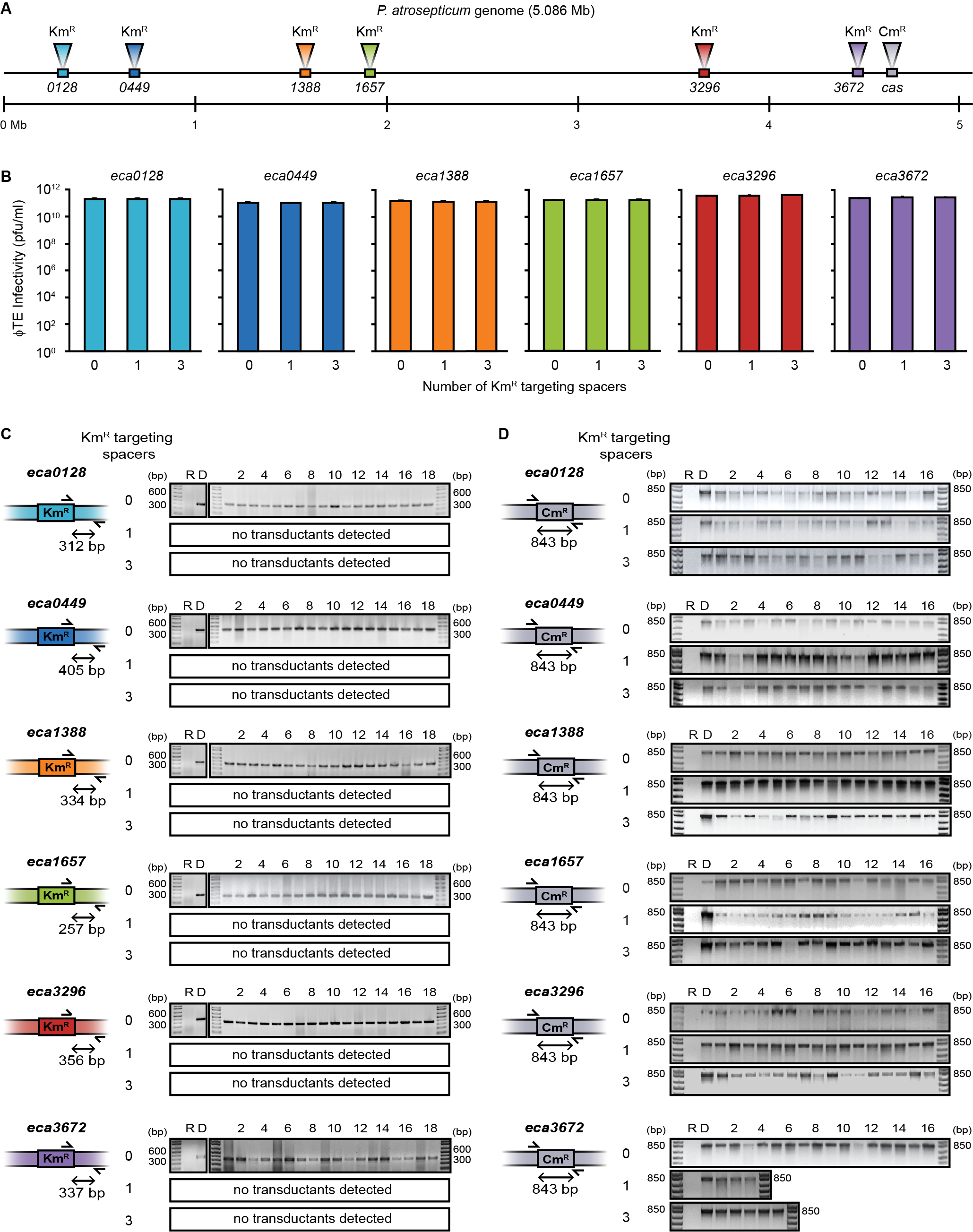

Supplement: FIG S2 [file mbo001183732sf2.tif]

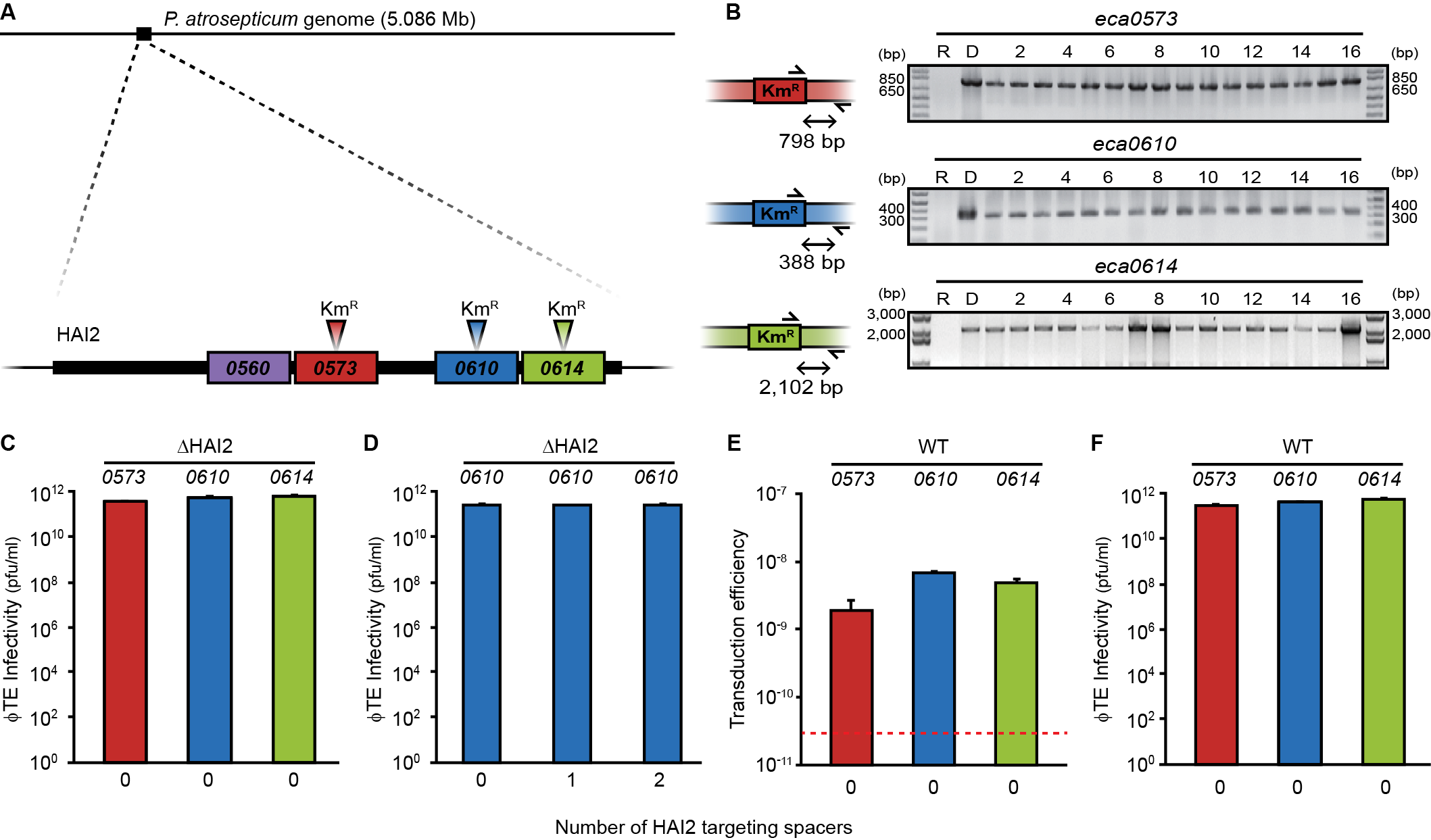

Supplement: FIG S3 [file mbo001183732sf3.tif]

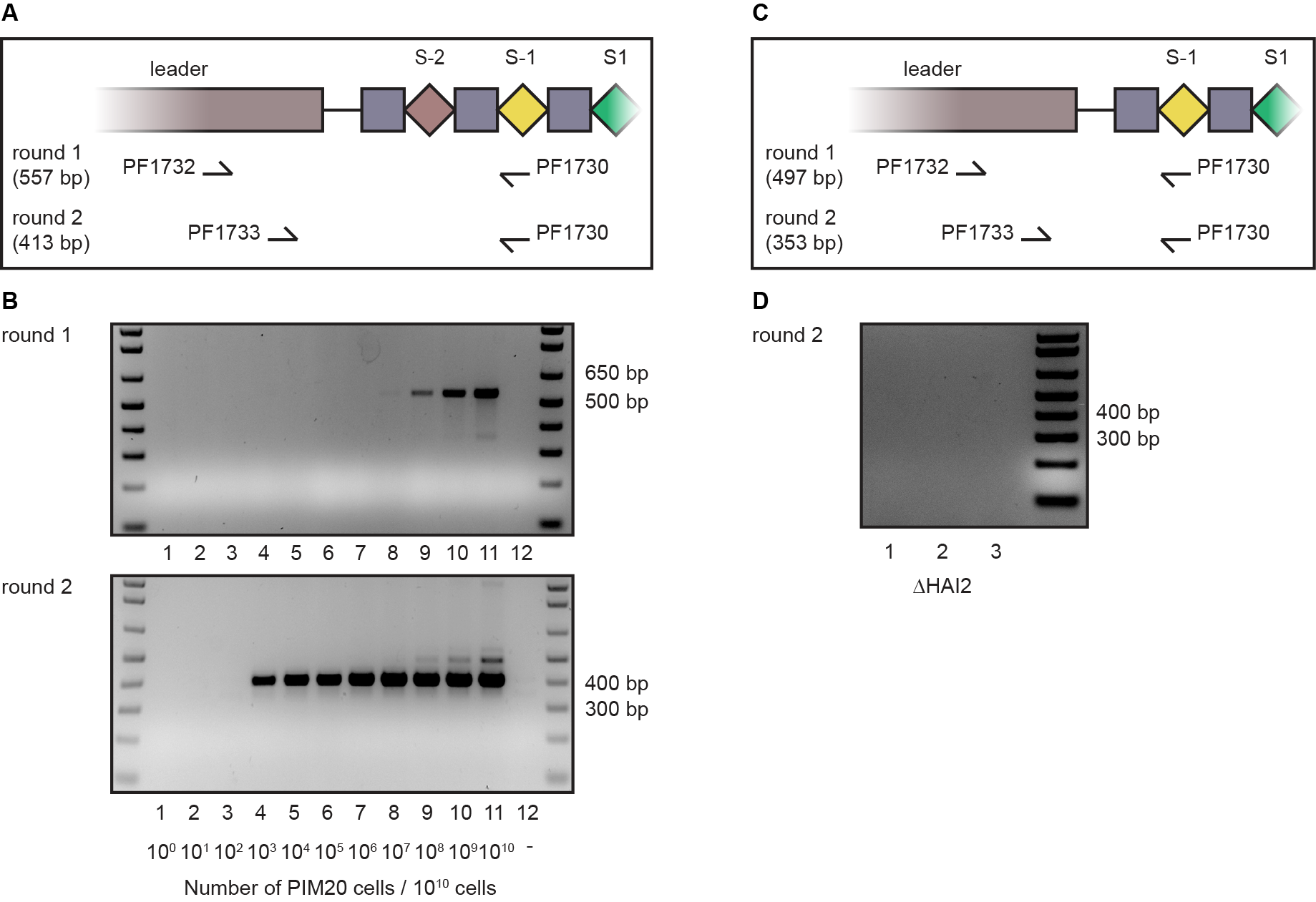

Supplement: FIG S4 [file mbo001183732sf4.tif]

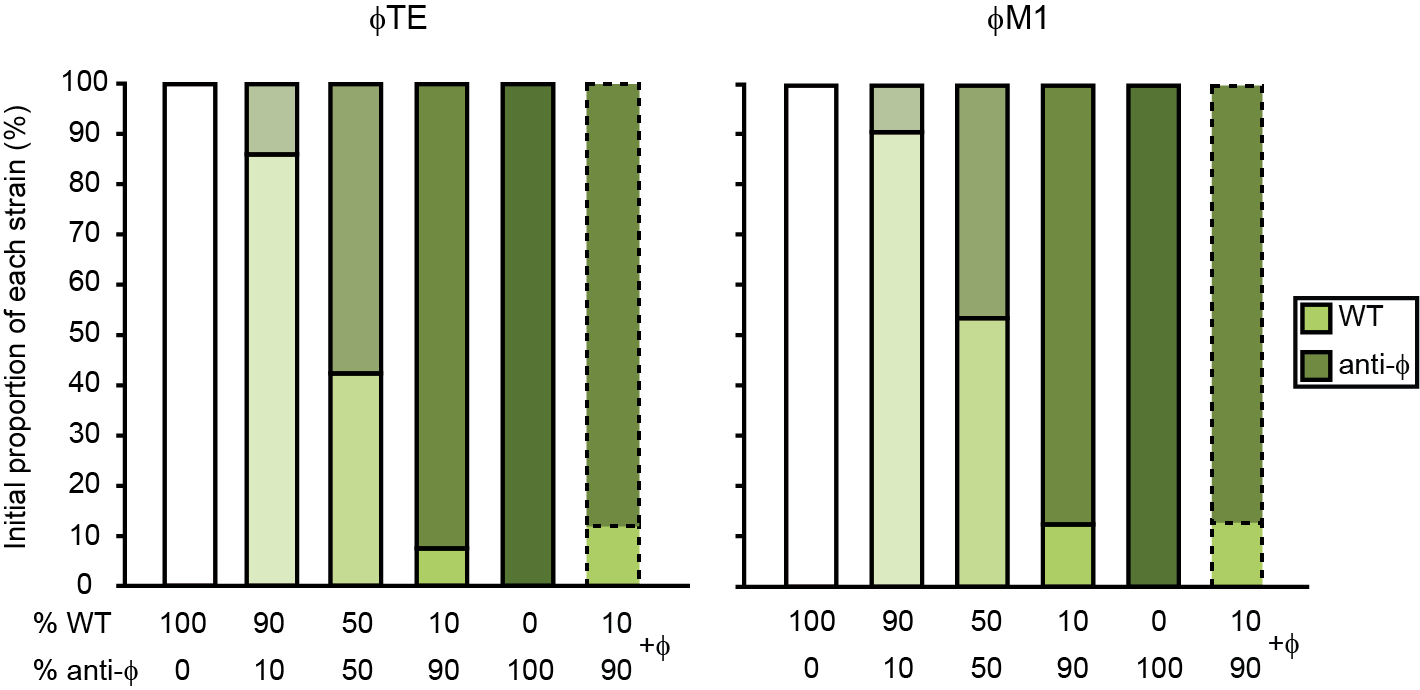

Supplement: FIG S5 [file mbo001183732sf5.tif]
