# Supplementary material for: CRISPR-Cas-Mediated Phage Resistance Enhances Horizontal Gene Transfer by Transduction
Source: mBio. 2018 Feb 13;9(1):e02406-17. doi: 10.1128/mBio.02406-17 (PMC5821089; doi:10.1128/mBio.02406-17)
Supplement: TABLE S1 [file mbo001183732st1.docx]

**Table S1**. Bacterial strains and plasmids used in this study.

| **Strain/Plasmid** | **Relevant Genotype/Phenotype** | **Reference** |
| --- | --- | --- |
| ***Pectobacterium atrosepticum* SCRI1043** | | |
| ΔHAI2 | ΔHAI2 (islandless deletion) | ([1](#_ENREF_1)) |
| PCF79 | Δ*cas*::*lacZcat* (*cas* operon deletion, *lacZ* translational fusion) in REM200 (*lacZ-* background), Cm^R^ | ([2](#_ENREF_2)) |
| PCF81 | Δ*expI*::*cat* (*expI* deletion), Cm^R^ | ([3](#_ENREF_3)) |
| PCF83 | *eca0128*::Tn-DS1028-*uidA*Km, Δ*cas*::*lacZcat*, Km^R^, Cm^R^ | This study |
| PCF84 | *eca0449*::Tn-DS1028-*uidA*Km, Δ*cas*::*lacZcat*, Km^R^, Cm^R^ | This study |
| PCF85 | *eca1388*::Tn-DS1028-*uidA*Km, Δ*cas*::*lacZcat*, Km^R^, Cm^R^ | This study |
| PCF86 | *eca1657*::Tn-DS1028-*uidA*Km, Δ*cas*::*lacZcat*, Km^R^, Cm^R^ | This study |
| PCF87 | *eca3296*::Tn-DS1028-*uidA*Km, Δ*cas*::*lacZcat*, Km^R^, Cm^R^ | This study |
| PCF88 | *eca3672*::Tn-DS1028-*uidA*Km, Δ*cas*::*lacZcat*, Km^R^, Cm^R^ | This study |
| PCF89 | *eca0573*::Tn-DS1028-*uidA*Km, Δ*cas*::*lacZcat*, Km^R^, Cm^R^ | This study |
| PCF90 | *eca0610*::Tn-DS1028-*uidA*Km, Δ*cas*::*lacZcat*, Km^R^, Cm^R^ | This study |
| PCF91 | *eca0614*:: mTn*5-gusA-pgfp21*, Δ*cas*::*lacZcat*, Km^R^, Cm^R^ | This study |
| PCF188 | Pba1043 with 3x anti-ɸTE spacers (CRISPR1+2) | ([4](#_ENREF_4)) |
| PCF190 | Pba1043 with 1x anti-ɸTE spacer (CRISPR1) | This study |
| PCF193 | Pba1043 with 1x anti-GFP spacer (CRISPR1) | This study |
| PCF254 | Pba1043 with 1x anti-ɸM1 spacer (CRISPR1) | This study |
| PCF256 | Pba1043 with 3x anti-ɸM1 spacers (CRISPR1+2) | This study |
| PCF287 | PCF400, *eca3672*::Tn-DS1028-*uidA*Km | This study |
| PCF288 | PCF332, *eca3672*::Tn-DS1028-*uidA*Km | This study |
| PCF326 | SCRI1043 with spontaneous Nal^R^ | This study |
| PCF332 | PCF188 with spontaneous Sm^R^ | This study |
| PCF400 | PCF256 with spontaneous Sm^R^ | This study |
| PIM06 | ΔHAI2 with 1x anti-*eca0560* spacer (CRISPR1) | ([1](#_ENREF_1)) |
| PIM17 | ΔHAI2 with 1x anti-*eca0560* spacer (CRISPR2) | ([1](#_ENREF_1)) |
| PIM18 | ΔHAI2 with 3x anti-Km spacers (CRISPR1) | ([1](#_ENREF_1)) |
| PIM20 | ΔHAI2 with 3x anti-Km spacers (CRISPR1+2) | ([5](#_ENREF_5)) |
| PIM28 | ΔHAI2 with 1x anti-Km spacer (CRISPR1) | ([1](#_ENREF_1)) |
| PIM31 | ΔHAI2 with 2x anti-Km spacers (CRISPR1+2) | ([1](#_ENREF_1)) |
| REM200 | *lacZ-* SCRI1043 derivative with *lacZ* amber mutation | R. Monson; unpublished |
| SCRI1043 | Wild type (WT) | ([6](#_ENREF_6)) |
|  |  |  |
| **Plasmids** |  |  |
| pPF189 (targeted) | pTRB30-derivative containing *eca0560*, Km^R^Wild type (WT) | ([1](#_ENREF_1)) |
| pTRB30 (non-targeted) | pQE-80L derivative with Ap^R^ replaced by Km^R^, Km^R^ | ([2](#_ENREF_2)) |
