# Supplementary material for: CRISPR-Cas-Mediated Phage Resistance Enhances Horizontal Gene Transfer by Transduction
Source: mBio. 2018 Feb 13;9(1):e02406-17. doi: 10.1128/mBio.02406-17 (PMC5821089; doi:10.1128/mBio.02406-17)
Supplement: TABLE S2 [file mbo001183732st2.docx]

**Table S2**. Oligonucleotides used in this study.

| **Name** | **Sequence (5'-3')** | **Description** | **Restriction site(s)** |
| --- | --- | --- | --- |
| PF118 | CGTAGAGGATCCCCGGG | LHS out mTn*5*-*gusA*-*pgfp21* |  |
| PF174 | CGTTAGAGTGATCGGGCTAC | F for CRISPR1 leader |  |
| PF175 | CAATGGCTCAGGGGATTC | R for CRISPR1 spacer 2 |  |
| PF176 | GGTAACTACCGTAAAATAGGAACG | F for CRISPR2 leader |  |
| PF177 | GCCTTTAAGCGCATGTCG | R for CRISPR2 spacer 2 |  |
| PF178 | CTTTAATAATCTGGTTGTTAGTGTG | F for CRISPR3 leader |  |
| PF179 | CCTCAGAAAGCCGACTTC | R for CRISPR3 spacer 2 |  |
| PF209 | TCGTCTTCACCTCGAGAAATC | F for pTRB30 MCS |  |
| PF210 | GTCATTACTGGATCTATCAACAGG | R for pTRB30 MCS |  |
| PF390 | AGGTGGATCCATGGATAACGCCTTTAGCC | F for *cas1* (*eca3679*) | BamHI |
| PF391 | AGGTCTGCAGCAGAATGTTCATCGCACTAC | R for *cas1* (*eca3679*) | PstI |
| PF432 | TTTGTCGACATACCGGGAAGCCCTGGG | F for *cat* (Cm^R^) | SalI |
| PF433 | TTTAAGCTTAGGCGTTTAAGGGCACCA | R for *cat* (Cm^R^) | HindIII |
| PF1212 | CGGGAATTCTCATGTTTGAC | RHS out DS1028-*uidA*Km |  |
| PF1216 | ATGGACTGGACTAATATTCCCC | F for *eca0610* on HAI2 |  |
| PF1219 | TTCTTGTACTGGTCGCGTTC | R for *eca0573* on HAI2 |  |
| PF1225 | TACGATGAAGCGAGAGCACA | F for *attP* (out RHS of HAI2) |  |
| PF1226 | CCGCCCTTTGTCGAAATTA | R for *attP* (out LHS of HAI2) |  |
| PF1227 | GATTCGTGGGGTGATTAAGG | F for *attB*, *phe-*tRNA |  |
| PF1228 | ACGTAGCTCAAGCCAGTCGT | R for *attB*, *phe-*tRNA |  |
| PF1243 | AAGATGCATTTTGTGCTTT | F for *eca0614* on HAI2 |  |
| PF1446 | GCTCCACCATCCCTCACT | F for *eca0560* on HAI2 |  |
| PF1447 | TGGTTAGCGTGGCTGG | R for *eca0560* on HAI2 |  |
| PF1457 | ATACGCGATTTCTAACGAATTATG | F for *eca1388* |  |
| PF1459 | CCTTTATTCATCAGCACAATACG | F for *eca3296* |  |
| PF1460 | GCTTAAACCACAGCATTAGTCG | F for *eca3672* |  |
| PF1573 | GAATTGTTATTCATTCCTCTCG | F for *eca0449* |  |
| PF1575 | AGACAATGTTTATTTTTATCACTACC | R for *eca0128* |  |
| PF1577 | AAATAGAGGAATACGAATATGATGC | R for *eca1657* |  |
| PF1730 | CGCTCCACCATCCCTCACT | R for HAI2-targeting spacer |  |
| PF1732 | GGAATATTCCAGTACAGAGACTGGTTTCC | F for CRISPR1 (nested1) |  |
| PF1733 | GCGTTATCCATAATGTATTTTCTTCCGTAA | F for CRISPR1 (nested2) |  |
